# Supplementary material for: Can the GRE predict valued outcomes? Dropout and writing skill
Source: PLoS One. 2022 Jun 1;17(6):e0268738. doi: 10.1371/journal.pone.0268738 (PMC9159619; doi:10.1371/journal.pone.0268738)
Supplement: Attachment — Submitted filename: response to PLOS reviewers.docx [file pone.0268738.s001.docx]

**Response to reviewers**

**Reviewer 1**

*p. 2 –…This discussion would be strengthened if the authors were able to briefly discuss the way that the development of the GRE minimizes the possibility of bias and/or share the results of studies establishing that group differences in GRE scores are not likely owing to bias.*

Added: “Trained fairness reviewers, including representatives of minority groups, review all GRE questions and statistical differential item functioning (DIF) procedures are used to identify any test questions that are unusually difficult (or easy) for a particular racial/ethnic or gender group.”

*p. 11 -- …While you did not use this analytic approach, it may be worth mentioning in the discussion that others such as Donald E. Powers 2004 paper in Journal of Applied Psychology: “Validity of Graduate Record Examinations (GRE) General Test Scores for Admissions to Colleges of Veterinary Medicine” have seen effects of range restriction for GRE scores.*

I now mention range restriction in the context of the Kuncel et al. study that was mentioned in the introduction but note that the correction can be problematic when the restriction is severe. Although Powers (2004) is a good study it is not as relevant because the restriction on both predictors and criterion was modest in the sample of veterinary schools.

Added: “Although some correction for the restricted range of the predictor scores is possible in correlational studies (e.g., Kuncel et al. [6]), the correction depends on fitting a regression line based on data that can be very sparse when the restriction is as severe as it was in this study.”

*p. 15. – I believe that a little more detail would be helpful to describe the process for establishing rater reliability. It appears that 214 of the 434 writing tasks were read by two readers (presumably 2 who were randomly paired from the pool of 12, and, I imagine, a separately randomly-assigned pair in each case, but that needs to be clarified, please. Also, while perhaps it should be, it is not clear to me how the inter-rater correlation was derived. (0.70). Please clarify.*

Added: “For each essay, the score from the first rater (randomly selected from the pool of 12 raters) was correlated with the score from the second randomly selected rater.”

*Conclusion: … Nonetheless, I would be interested in the authors discussing why they chose not to compare group differences, given the purpose of the paper.*

Sample sizes were too small to compare group differences.

Added in the discussion: “Additional research is needed to better understand the role of GRE scores in less selective programs, and to evaluate possible differential effects in racial/ethnic and gender groups that could not be evaluated in this study because of the limited sample sizes for these groups.”

**Reviewer 2**

*1) …For instance, noting that the average GRE-Q score of completers in [3] was 723 means little without knowing where that sits in context (a quick search of ETS indicates that’s around a 65th percentile, which some faculty likely would not consider a “high” GRE-Q score).*

Added that 723 is about 65^th^ percentile. Also toned down comment on “high” scores on the GRE to say simply “none of the students with three or more publications had low GRE-Q scores.”

*2) A reference on range restriction (e.g.* [*https://arxiv.org/abs/1709.02895*](https://arxiv.org/abs/1709.02895)*) might help readers unfamiliar with these issues.*
Referred reader back to the Kuncel study mentioned in the introduction in the context of range restriction.

*3) Authors should consider citing Kuncel et al. (2001), which actually corrects for range restriction issues and is often cited on the ETS website, but finds no significant correlation between GRE-Q (or -V) and PhD completion in their meta-analysis. How do the authors reconcile this result with their current manuscript?*
Kuncel was cited in the introduction, but it does not deserve too much attention. Many of the studies in the meta-analysis are over 30 years old when the test itself as well as the populations taking it were quite different than they are today. This why the current study was needed.

*4) A follow-up to Ref [8] (*[*https://doi.org/10.1103/PhysRevPhysEducRes.17.020115*](https://doi.org/10.1103/PhysRevPhysEducRes.17.020115)*), in general, finds the same basic conclusions as this manuscript (that GRE-Q is correlated with PhD completion), and addresses several concerns laid out in [9]. As such it is likely worth citing.*

The follow-up states, “Students’ undergraduate GPA (UGPA) and GRE Physics (GRE-P) scores are small but statistically significant predictors of graduate course grades, while GRE quantitative and GRE verbal scores are not,” but corrects only a few of the problems outlined in [9], so it does not seem to be worth citing.

*5) Lastly, the literature review is entirely dedicated to studies most related to Study 1, in particular the existing literature surrounding GRE-Q. Something needs to be said about Study 2 in order to frame it within a larger context. Has there been previous work on standardized writing (on SATs? APs?) and success?*
Added: “In addition to program completion, another valued outcome for graduate programs is writing skill. Strong writing skills are required in many graduate courses and in all doctoral programs with a thesis requirement. There is evidence that the GRE Analytical Writing test predicts graduate grades across a number of graduate programs. Indeed, in a comprehensive study using data from over 25,000 students from 10 universities in the Florida state system the GRE Analytical Writing (GRE-AW) test was a significant predictor of the graduate grade point average across a number of different programs [6]. GRE-AW was frequently a better predictor than either the GRE-V or GRE-Q scores, perhaps surprisingly predicting grades in master’s engineering programs and biomedical PhD programs better than predictions from GRE-Q. Because many factors in addition to writing skill are important in determining the overall grade point average, this study could not provide a direct link between GRE-AW and writing demands in graduate courses.”

*6) The authors must either discuss in more detail the data collected on the History and Psychology programs or entirely remove the comment referring to their elimination from the study. Presumably the History majors had the largest spread of GRE-Q scores (as a non-STEM field), yet they all graduated anyway? Was this just a small N problem or are we seeing essentially no correlation between GRE and completion within history majors (since they’re all graduating independent of score)? This deserves further comment.*

Because our plan was to analyze results in four programs, dropping mention of the history and psychology does not seem to be appropriate. It is not exactly a small N problem but is a problem because of the very small N of students who drop out of these programs. It is not possible to predict dropout when students are not dropping out.

*7) While it is understandable to want “intuitive” results, some readers will immediately note that by splitting data into quartiles, the authors are “throwing away” some information in the data. Consider including both the correlation and 2x2 tables even if some readers may "misinterpret" correlations.*

Added correlation.

*8) With regard to interpretability, the audience of faculty on admissions committees for whom this paper would most likely be targeted may be unfamiliar with logistic regression using independent variables that have been split into quartiles. A brief comment on what the estimates in Tables 2/4/5 mean in terms of practical score changes and probability of completion would be helpful.*

Note that the logistic regression used the original data before it had been split into quartiles.

Added: “Similar to an ordinary least squares regression, the logistic regression provides an estimate of the importance and statistical significance of each predictor in the equation but is appropriate when the criterion is dichotomous (0-1 drop out or stay).”

*9) As mentioned in comment 5), Study 2 lacks the same context as Study 1. As it stands, essentially the entire discussion in the introduction is dedicated to the research landscape as it pertains to GRE-Q, with little to no mention of GRE-V or writing. Hence Study 2 feels only loosely connected to the rest of the paper.*

See response to comment 5.

*10) In the same spirit as the previous comment, Study 2’s methods and data should be discussed in more detail. For example,
- How many students were included? What are the demographics of the students who submitted writing samples? What are their majors? What can the study say about majors not included?
- What are the demographics of the faculty who graded the responses? Do these align, and does it matter?
- For instance, the rubric appears to be constructed so that faculty from any discipline can grade papers from any other discipline (ie, a psychology faculty member might grade a history paper and evaluate its issue and argument) - is this justified? Can a faculty member determine whether an argument is good when it is out of disciplinary context? Perhaps, but this decision is not discussed. What information was lost by stripping disciplinary context?
- One possible avenue to explore this is to examine the scores given by the students’ instructors in comparison to those given by the independent graders.
- Have previous studies used a similar methodology to evaluate written work?
- What do the distributions of scores look like on the graded papers? What about the original grades? What do these say about the sample of students?
- Were there specific requirements for a paper to be “deemed scorable” by the scorers?
Some of these questions have been addressed, but data was not available for many of them. The similarity of the approach taken here to previous research [15] was acknowledged.*

Added: “Therefore, the approach taken followed procedures used in a previous study of student writing in realistic classroom contexts, and the scoring approach also closely matched the procedures in the previous study [15].”

The need for additional work in this area was also acknowledged.

Added: “Future research should provide more detailed analyses related to the prediction of writing skills. Specifically, the match between the rater’s area of expertise and the assigned writing task should be explored, and with a larger sample analyses within specific program areas should be feasible.”

*… Amending the abstract to include details of the study (e.g. 4 programs studied at 2 universities, N = students, GRE-Q found significantly associated with completion for one of the programs) avoids any possible obfuscation of the findings.*

Amended abstract: “…Two studies are included. The first study used data from chemistry (N=320) and computer engineering (N=389) programs from a flagship state university and an Ivy League university to demonstrate the ability of the GRE to predict dropout. Dropout prediction for the chemistry programs was both statistically and practically significant for the GRE quantitative (GRE-Q) scores, but not for the verbal or analytical writing scores. In the computer engineering programs, significant dropout prediction by GRE-Q was evident only for domestic students. The second study showed the relationship of GRE Analytical Writing scores to writing produced as part of graduate school coursework by 217 students.

*Put another way, why should faculty believe that a students’ ability to do high school math questions make them more able to complete a PhD?*

Although GRE-Q does not require computational skills beyond high school math, the reasoning required is far beyond “ability to do high school math.” If only high school knowledge were required half of the examinees would not have scores below 154 and GRE-Q could not predict either grades or dropout.

*Or are other variables confounding this relationship? Does it matter, so long as the test is indicating whether students will drop out?*

There may be many intervening variables, but this does not alter the conclusion that GRE-Q scores are a useful indicator of students who are more likely to drop out.

*…the authors must reconcile statements such as “Although there is ample evidence that the scores can predict graduate school readiness as indexed by grades in the first one or two years (e.g., [5,6]) mere grade prediction is of limited value.” with the fact that Study 2 uses writing samples produced in graduate coursework. Is that not merely a prediction of graduate grades?*

No; writing is just one aspect of graduate grades and this study permits focus on just the writing skill component.
