# Supplementary material for: Can the GRE predict valued outcomes? Dropout and writing skill
Source: PLoS One. 2022 Jun 1;17(6):e0268738. doi: 10.1371/journal.pone.0268738 (PMC9159619; doi:10.1371/journal.pone.0268738)
Supplement: Attachment — Submitted filename: response to plosone reviews April.docx [file pone.0268738.s002.docx]

Response to reviewers (April 2022)

*the author response to the reviewers regarding rater reliability does not appear in the new manuscript*

added: For each essay, the score from the first rater (randomly selected from the pool of 12 raters) was correlated with the score from the second randomly selected rater. The inter-reader correlation for a single essay was .70.

*Reading the paper, there is an unsettling dissonance. The authors criticize former studies for focusing on highly selective institutions and programs (with slight score variation).* ). *Yet, their study is based on data from highly selective institutions*

This was addressed by modifying the methods and materials for Study 1 as follows:

We had requested data from graduate programs representing a variety of selectivity levels but were ultimately successful in obtaining data from only two universities. GRE scores and program completion data were obtained from four highly selective PhD programs at a large flagship state university and at a highly selective Ivy League university. We understood that finding significant relationships to dropout in highly selective programs would likely be challenging, but even in these selective programs there was some variation in GRE scores, albeit near the top of the score scales.

*The analysis itself is ok. It would be good to explain why picking ML over other options for the logistic regression. And as there are a growing number of studies that turn to machine learning models that are ideal for prediction purposes (random forests is but one option), this statistical approach seems valid but outdated….* *I understand that you don’t have the data on ethnicity, gender, and so forth, but even just using a more fine-tuned classification will do….explain the limitation and decision to focus solely on GRE in the regression*

In the conclusion we acknowledged that other analytic approaches were possible, and by implication why we chose not to use some covariates (e.g., undergrad GPA) that might make sense in other contexts but were less relevant to the current context.

More elaborated regression models, or random tree models, that account for additional predictors or covariates such as undergraduate grades or socioeconomic statue should also be considered but note that such variables are often difficult or impossible to interpret in populations with large numbers of international students with undergraduate grades on different scales and with socioeconomic indicators that may have different meanings internationally.

*I was surprised to see no reference to Bowen and Bok work. It is also possible to think about the connection between your argument (which is really somewhat of a “don't throw the baby out with the bathwater”) and the ongoing debates about affirmative action.*

Bowen & Bok reference added in the Conclusion:

With ample evidence available on the value of enrolling a diverse array of students [17], it would be unfortunate to ignore any measures that could help with this effort.

*Page 1: Please, also include results of the second study in the abstract*

Added to abstract: . In the second study, GRE Analytical Writing scores for 217 students were related to writing produced as part of graduate school coursework and relationships were noted that were both practically and statistically significant.

*Page 5: [… GRE because of a number of technical issues in the analysis [9].] > List the technical issues briefly.*

Added: ]. Specifically, the Abstract of this critique noted, “The paper makes numerous elementary statistics errors, including introduction of unnecessary collider-like stratification bias, variance inflation by collinearity and range restriction, omission of needed data (some subsequently provided), a peculiar choice of null hypothesis on subgroups, blurring the distinction between failure to reject a null and accepting a null, and an extraordinary procedure for radically inflating confidence intervals in a figure.”

*Page 8: Please, mention that, now, you also include correlation coefficients*

Added: We computed the correlation of GRE scores to dropout (0-1),

*Page 10: The numbers of students included in the study on page 9 (117 from a flagship state university and 198 from an Ivy League university) sum up to 315 and not to 320 as indicated in the caption of table 2. Please check and correct the numbers if necessary.*

Fixed

*Page 10: There is a space missing in the last paragraph: “were 164 and170”.*

Fixed

*Page 12: In the result section you claim “Results from the chemistry programs clearly indicate that GRE-Q scores can be effective in identifying students most likely to drop out”. Given that you show that 30% of students with a low GRE-Q score drop out and that 14% with a high GRE-Q score drop out, I would rather suggest to claim that “… in identifying students with a higher likelihood to drop out”.*

Fixed: … in identifying students with a higher likelihood to drop out.

*Page 21, reference 5: no space in “meta-analytic”.*

? I did not see any extra space. Spelling exactly as in the original reference.

*Page 22, reference 12: “;” not in italic font & wrong dash in page numbers.
Page 22, reference 13: “Rubin, D” instead of “Rubin ,D” & “, 74; 1982,” not in italic font*

fixed
